# A Further Improvement in the Room-Temperature Formability of Magnesium Alloy Sheets by Pre-Stretching

**DOI:** 10.3390/ma13112633

**Published:** 2020-06-09

**Authors:** Umer Masood Chaudry, Kotiba Hamad, Jung-Gu Kim

**Affiliations:** School of Advanced Materials Science & Engineering, Sungkyunkwan University, Suwon 16419, Korea; umer@skku.edu

**Keywords:** AZ31-0.5Ca, magnesium, pre-stretching, electron back scattered diffraction, formability

## Abstract

Pre-stretching experiments were carried out on AZ31–0.5Ca magnesium alloy to alter the microstructure and texture for enhancing room-temperature formability. Compared to as-received alloy, the formability of a 5%-stretched sample was improved by 15%. This was attributed to enhanced strain hardening capability related to the weakening of basal texture and less homogeneous microstructure. In addition, in-grain misorientation axis analysis performed on the samples (as-received and stretched) also confirmed the higher activity of the non-basal slip systems in the 5%-stretched sample.

## 1. Introduction

Magnesium (Mg) alloys have been one of the most attractive metallic materials based on the multiple advantages they offer. Mg alloys possess high specific strength and low density, which make them the most suitable candidate to be used in several fields. The vast applications of Mg, on the other hand, are restricted, and this is attributed to its room-temperature (RT) intrinsic brittleness caused by the insufficient deformation mode. This, in turn, is due to its HCP structure (hexagonal close packed). For uniform deformation in polycrystalline metals at room-temperature, five independent slip systems are required. However, pure Mg just has three slip systems from basal <a> slips, which cannot accommodate the tensile strain along the *c-axis* [1]. Additionally, due to the large gap between the critical resolved shear stress (CRSS) of basal and non-basal slips (~1 in basal to 40 in prismatic), cracks can be initiated, leading to inferior mechanical properties [2]. In addition, rolled Mg alloy sheets with strong texture lead to larger anisotropy and lower stretch formability. Alloys with strong basal texture need to accommodate strain along the thickness direction by secondary deformation modes, in which material undergoes in-plane tensile stress [3]. Even though huge efforts have been put towards enhancing the performance of Mg alloys, these are restricted by the huge cost and low productivity. Mg can be made highly formable by specific-solute alloying, which can decrease the first intrinsic stacking fault energy (I_1_SFE) [4], delay the pyramidal-to-basal transition (PB transition) [5] or enhance the cross-slip of pyramidal II dislocation [6]. In addition, a homogeneous distribution of second-phase particles is very critical, otherwise inhomogeneous distribution can result in premature failure in second-phase-free and second-phase-rich regions. Secondly, properties can be enhanced by the modification of microstructure and weakening of strong basal texture, which will result in a high Schmid factor and high resolved shear stress in the basal plane.

It is well known that the formability of Mg can be enhanced by weakening of the basal texture (0002) [7]. Kang et al. [8], reported that an increase in grain size and randomization of texture resulted in a higher work hardening exponent, which lead to improved formality in Mg alloys. In addition, Song et al. [9] introduced the contraction twins in AZ31 alloy by pre-cold-rolling to a 10% thickness reduction and recorded a 65% increase in formability of cold-rolled alloy as compared to as-received alloy. This improvement was mainly explained based on the role of the contraction twins in inducing the nucleation and growth of randomly orientated recrystallized grains. Furthermore, Weijun et al. [10] observed a 50% improvement in stretch formability of AZ31 by introducing $\left\{10\bar{1}2\right\}$ tension twins by in-plane compression. The improved room-temperature stretch formability was attributed to the formation of tensile twins in the pre-twinned region. Zhang et al. [11], carried out the pre-stretching process on AZ31 alloy and found a remarkable increase of 65% in the stretch formability of pre-stretched alloy as compared to the as-received alloy. The significant increase in stretch formability was attributed to a smaller anisotropy (r-value) and larger strain hardening exponent related to texture and grain size effect. In addition, Chino et al. [12], carried out room-temperature tensile tests and stretch-formability experiments on rolled AZ31 Mg alloy and studied the effects of the grain size. The stretch formability was found to be in direct relation to the grain size which was attributed to the lattice rotation by the twins under biaxial tensile stress. In conclusion, microstructural and textural modifications were very significant in enhancing the performance of Mg alloys. Recently, a new Mg alloy was introduced by the addition of 0.5 wt.% Ca to commercial AZ31 (Easy-form (E-form)) with higher deformability. As reported by our previous studies [13,14], E-form alloy showed improved room temperature formability as compared to AZ31. The formation of (Mg,Al)_2_Ca particles contributed towards weakening of the basal texture. In addition, the CRSS calculations evaluated by the viscoplastic self-consistent (VPSC) model and in-grain misorientation axis (IGMA) analysis of E-form alloy confirmed that formation of such particles enhanced the activity of non-basal slip systems [15]. The aim of this study is to further improve the stretch formability of the E-form alloy by modification of the structure and texture, and/or by introducing tension twins, which have a significant role in enhancing the formability of Mg alloys [16,17,18,19,20].

## 2. Experiments

In the present work, E-form (AZ31–0.5Ca) sheets were provided by POSCO (S.Korea). For stretching experiments, as-received E-form alloy sheets with composition shown in Table 1 were cut into 120-mm (length) × 40-mm (width) specimens along the rolling direction (RD). Pre-stretching was carried out on a CMT6305-300KN testing machine (Tokyo, Japan)) along the rolling direction at room temperature. The specimens were stretched to 5%, 10% and 15% at the strain rate of 10^−3^ s^−1^. For microstructural and textural evolution, electron-backscattered diffraction (EBSD) was performed. For the EBSD observations, pre-stretched specimens cut from the ND-RD plane were ground and then polished by a cross-sectional polisher (Hitachi IM4000, Tokyo, Japan) A scanning electron microscope with a field-emission gun (Hitachi S-4300 FESEM, Tokyo, Japan) was then employed to observe the polished samples and a TSL OIM 6.1.3 was used to analyze the data. For accurate measurement, a small step size (0.05 μm) was used during EBSD scanning. Tensile tests were performed at room temperature on pre-stretched samples with gauge length of 25 mm, width of 6 mm and thickness of 1 mm. For Erichsen cupping tests, circular blanks with a diameter of 35 mm were machined from the pre-stretched samples. An Erichsen test was conducted using a hemi-spherical punch at a punch speed of 0.1 mm/s with (the width of a specimen panel is 13–90 mm).

## 3. Results and Discussion

### 3.1. Structure

**Figure 1:**
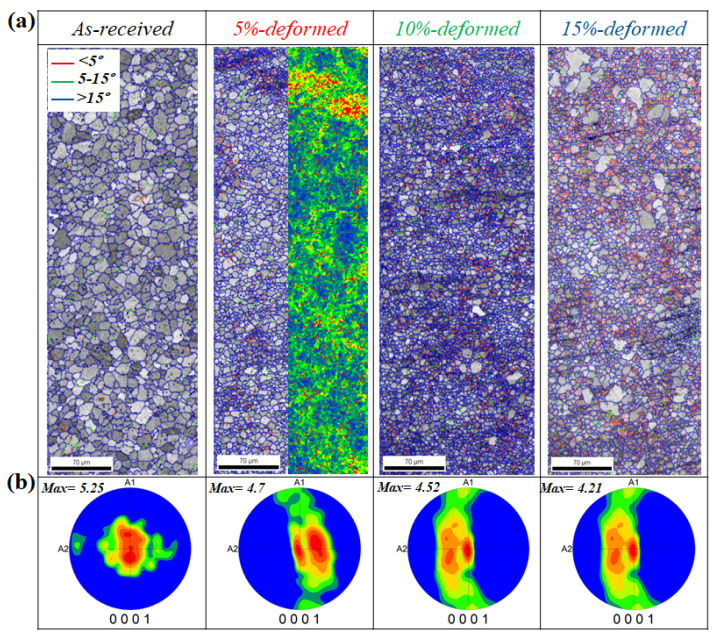
(**a**) Image quality (IQ) maps and (**b**) pole figures (PFs) of the as-received and stretched (5%, 10% and 15%) samples. Here, A1 and A2 are rolling direction (RD) and transverse direction (TD) of the samples, respectively. The KAM map of the 5% sample is inserted to show the GNDs.

Figure 1a shows the image quality (IQ) map superimposed with the grain boundaries of as-received and stretched samples. It is evident from the IQ map that the as-received alloy consisted of an equiaxed fine-grained (7.9 µm) structure. The IQ map of 5%-stretched sample revealed a less homogeneous microstructure, containing randomly distributed fine and coarse grains. The IQ map of 10%-stretched sample showed a microstructure mostly composed of fine grains, suggesting that further deformation leads to a decrease in overall grain size, where a higher proportion of low angle grain boundaries (misorientation angle (𝜽) < 15°) can be noticed (represented in green color) as compared to the 5%-stretched sample. In general, dynamic recrystallization (DRX) is used to explain the formation of fine-grained structure during plastic deformation of Mg-based materials. In order to activate the DRX, Mg materials should be stretched at elevated temperatures, and this can be enhanced using high-strain-rate stretching. However, in the present experiments, a room-temperature deformation was employed, and, accordingly, the formation of the finer-grained structure cannot be fully figured out based on the occurrence of DRX; especially, no clear microstructural evidence was found through EBSD measurements. The evolution of the finer structure during the stretching of this alloy might be related to the dislocation accumulation near the grain boundaries, which leads to formation of a substructure. This is shown through the kernel average misorientation (KAM) measurements conducted by EBSD, as shown by the KAM maps of the 5% sample inserted in Figure 1. As is well established, the KAM value shows the presence of geometrically necessary dislocations (GND) that are related to local lattice distortion or localized deformation. In this regard, a microstructure can be regarded as a strain-free structure if its average KAM value is less than 1° [21]. Further deformation to 15% leads to a higher proportion of low-angle grain boundaries (𝜽 < 15°, shown in green color), where a simultaneous increase in the grain growth energy resulted in a slight increase in grain size, as evident from Figure 1a.

Figure 1b shows the (0001) PF of the as-received alloy and the samples stretched to 5%, 10% and 15%. It is evident that stretching experiments led to the weakening of basal texture, where the lowest intensity, 4.21 was received for the sample stretched to 15%. In addition, the basal poles were found to be broadly distributed along the RD. This indicates that the *c-axis* of grains in the stretched samples was either distributed along the transverse direction or parallel to the normal direction [22]. Generally, a strong basal texture, in which the *c-axis* is perpendicular to the RD, is evolved in Mg-based materials after primary processing, as shown by the pole figure of the non-deformed sample (Figure 1). In the simple model that describes lattice rotation during tensile deformation of Mg materials, the c-axis of the crystal can be rotated along the tensile axis to the TD-RD plane of the sample.

**Figure 2:**
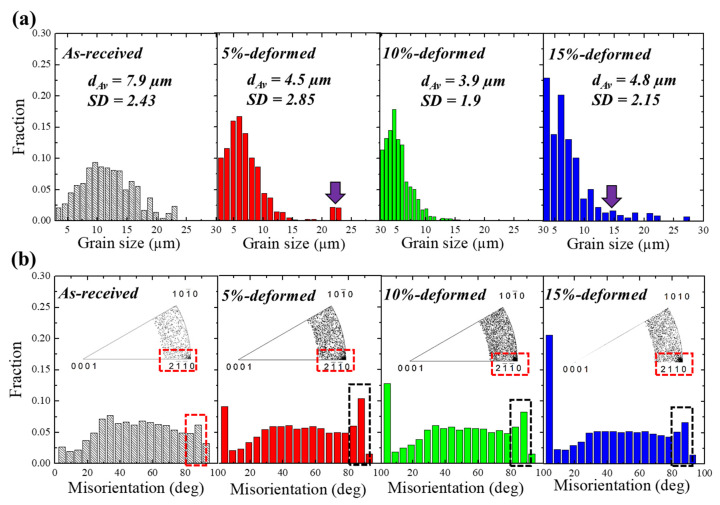
(**a**) Grain size distributions and (**b**) misorientation profiles of as-received and pre-stretched (5%, 10% and 15%) samples. The figure inserted in Figure 2b is the misorientation distribution function of the samples.

EBSD analysis was carried out to confirm the effect of pre-stretching on microstructural features of samples including microstructural homogeneity, grain size and misorientation distribution. Figure 2a shows the fractional distribution of the grain size of as-received and stretched samples. It is clearly seen that the as-received alloy showed a homogenous distribution with average grain size and standard deviation (SD) of 7.9 µm and 2.43, respectively. As the sample was stretched to 5%, the microstructure was observed to be less homogeneous as compared to as-received sample, where an SD of 2.85 was measured for the 5%-stretched sample. Additionally, some fractions of coarse grains with average grain sizes of 22 µm were also observed for the 5%-stretched sample, as shown by the purple row inserted in the distribution profile of the 5%-stretched sample. The SD value was found to be the highest for 5% among all the samples (1.9 and 2.15 for 10%- and 15%-stretched samples, respectively), which confirms the less homogeneous structure of this sample as compared to other samples (as-received and pre-stretched). The grain size distribution of the sample stretched to 10% showed the presence of a high proportion of fine grains with an average grain size of 3.9 µm. As aforementioned, further deformation beyond 5% can result in the initiation of dynamic recrystallization which can lead to an overall decrease in grain size. For 15%-stretched sample, the deformation leads to evolution of a higher fraction of dynamically recrystallized grains with a lower overall grain size and a simultaneous increase in the grain growth energy at higher strain, leading to an increase in grain size [23].

Figure 2b shows the misorientation angle distribution of the as-received and pre-stretched samples (5%, 10% and 15%). It is evident from Figure 2b that the misorientation distribution of the as-received sample showed a lower fraction of grain boundaries with a low angle of misorientation: those with angles smaller than 15° (low-angle grain boundaries (LAGBs)). In addition, a higher fraction of high-angle grain boundaries (HAGBs) with misorientation angles bigger than 15° was observed in this sample. By stretching, the fraction of LAGBs was dramatically increased, and this was at the expense of decreasing the fraction of HAGBs, where LAGBs vs. HAGBs fractions of 0.068 vs. 0.932, 0.135 vs. 0.865, 0.171 vs. 0.829 and 0.251 vs. 0.749 were measured for the as-received, 5%,- 10%- and 15%-stretched samples, respectively. Furthermore, the fraction of the boundaries related to the evolution of tension twins (TT; $\langle \bar{1}011\rangle \left\{10\bar{1}2\right\}$) at ~86° slightly increased after the 5% stretching as compared to the as-received sample (0.06 vs. 0.10). However, the 10%- and 15%-stretched samples showed smaller fractions of TT-related boundaries (0.08 and 0.06 for 10%- and 15%-stretched samples, respectively). Although the samples were subjected to different straining conditions at room-temperature, the fractions of such boundaries (TT-related boundaries) were not greatly affected. This, additionally, was confirmed by misorientation distribution function (MDF) analysis, as shown by the figures embedded in the misorientation profiles of the sample (Figure 2b). These figures show that, after stretching, the number of points concentrated near $\langle 2\bar{1}\bar{1}0\rangle$ slightly increased, but they remained nearly unchanged by increase in strain. It is well established that, in Mg alloys, as hard-to-deform materials, TTs can act as a secondary deformation mechanism in addition to basal slips, leading to accommodation of the extension along the *c-axis*, and hence, contributing to room-temperature plasticity [19]. In the present work, on the other hand, a smaller number of TTs were observed in the AZ31–0.5Ca alloy as compared to those observed in other Mg alloys subjected to similar processing conditions [11,17]. For example, Zhang et al. [11], carried out pre-stretching on AZ31 and found a significant effect of applied strain on the propensity of twins. Junjie et al. [17] carried out pre-hardening experiments on AZ31 alloy and observed a significant improvement of mechanical properties in pre-hardened alloy as compared to AZ31, which was attributed to the evolution of tension twins. Interestingly, in the present alloy (AZ31-0.5Ca), the evolution of a higher fraction of LAGBs by stretching, which was at the expense of HAGBs diminishing, was more observable as compared to TT evolution.

**Figure 3:**
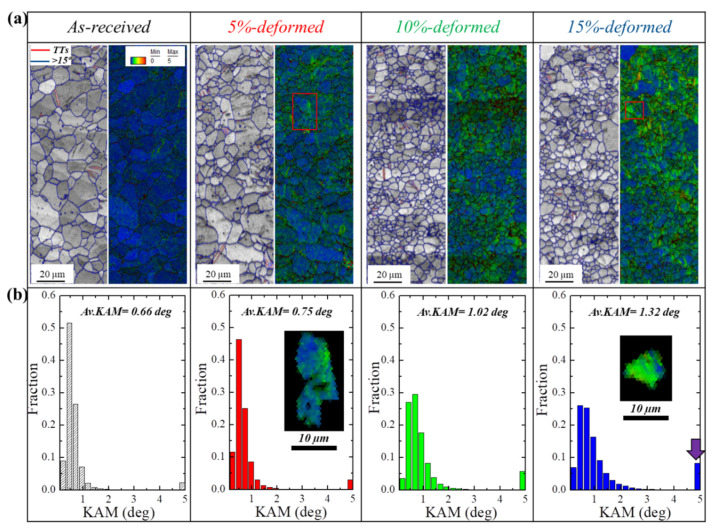
(**a**) Image quality with tension twins and kernel average misorientation and (**b**) kernel average misorientation profile of the of the as-received and stretched (5%, 10% and 15%) samples. The figures inserted in Figure b show separated grains taken from indicated areas in Figure 3a.

Figure 3 shows the high-magnification IQ maps, kernel average misorientation (KAM) and KAM profiles taken for the samples (as-received and pre-stretched). IQ maps were found to be in agreement with the misorientation distribution, where no significant effects on evolution of twins as a result of applied strain were observed (TTs shown by red color in IQ maps. It is well known that, due to the high CRSS of non-basal slip systems as compared to basal slip, the RT plasticity of pure Mg is mainly governed by the operation of basal slip systems (three systems) [24,25]. Accordingly, to satisfy the von Mises criterion, more modes will be needed, and this can be achieved through the operation of twins [26]. Based on this idea, the fewer fractions of TTs observed for the stretched samples in the present work can be attributed to the contributions of non-basal slip systems (prismatic and/or pyramidal) to the room-temperature plasticity of this alloy (AZ31-0.5Ca). To evaluate such contributions by slip systems to the plasticity process of this alloy, KAM was calculated from EBSD analysis. As is well established, the KAM value shows the presence of geometrically necessary dislocations (GND) that are related to a local lattice distortion or localized deformation. In this regard, a microstructure can be regarded as a strain-free structure if its average KAM value is less than 1° [27,28]. Hence, the KAM method is extensively used to determine the strain energy in terms of local distribution, which is internally stored in the material as a result of deformation. KAM maps and KAM profiles of as-received and stretched samples presented in Figure 3a,b revealed that as-received sample was strain-free, where an average KAM value of 0.66 was recorded in this sample. It is well established that, when material undergoes plastic deformation, the compatibility of plastic strain between neighboring grains can be achieved through the generation of additional stresses at GBs; this usually leads to increasing GNDs near the GBs [29]. This behavior is clearly seen in the 5%-stretched sample, where a higher average KAM value (0.75) was recorded, and, more importantly, the areas with high KAM values were recorded near GBs, as shown by the single grain inserted in the KAM profile of the 5%-stretched sample. A further increase in applied stress resulted in a higher accumulation of internal stored energy in the material; consequently, the average KAM values were found to be increasing in the 10%- and 15%-stretched samples (1.02 and 1.32, respectively). In addition, the high-KAM areas were uniformly distributed throughout the grain, as shown by the single grain inserted in the KAM profile of the 15%-stretched sample.

Such behavior, where slip systems operate more dominantly as compared to twins, noted in the present alloys AZ31–0.5Ca (Figure 3b), is explained based on the role of Ca addition in altering the activity of slip systems [13,15,30]. Yuasa et al. [31], for example, studied the effects of 0.06 wt.% Ca additions on the room-temperature formability of the Mg–1.5Zn alloy. The reported results revealed that the enhanced formability in the alloy containing 0.06 wt.% Ca (Mg–1.5Zn–0.06Ca) was mainly related to the operation of prismatic slip. This was figured out based on the role of dissolved Ca atoms in reducing the generalized stacking fault energy in this alloy. In addition, VPSC calculations and EBSD-IGMA carried out in our previous work also confirmed the increase in the activity of non-basal slip systems, which was attributed to the formation of (Mg,Al)_2_Ca particles due to 0.5 wt.% Ca addition. These particles were found to reduce the relative CRSSs (τ_Prismatic_/τ_Basal_) [15], thus leading to the higher activity of non-basal slip systems. Recently, the role of Ca has also been studied by Kim et al. [32], who designed a new Mg alloy (AZMX3110: Mg–3Al–1Zn–1Mn–0.5Ca), which showed remarkable improvement in strength and ductility at room temperature. This simultaneous improvement in properties was attributed to the new alloying elements (Mn and Ca) which induced precipitation and maximized the segregation of the other alloying elements.

### 3.2. Formability and Mechanical Performance

**Figure 4:**
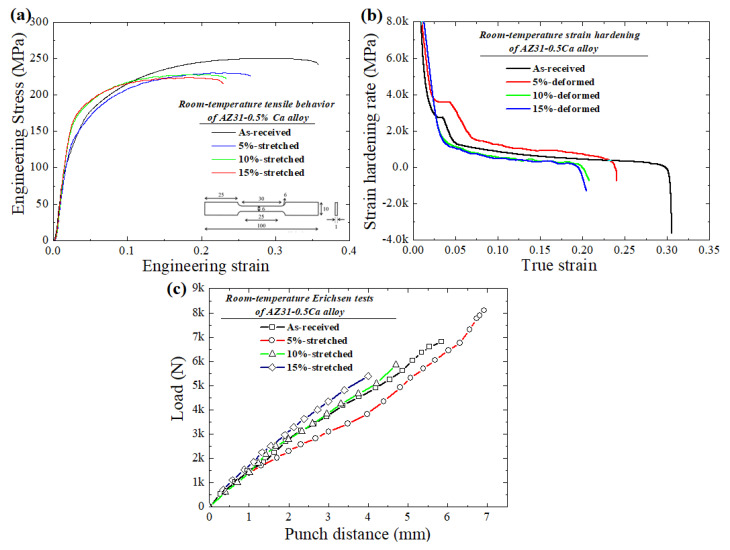
(**a**) Room-temperature tensile curves (figure inset in (**a**) shows the dimensions of the tensile samples); (**b**) strain hardening behavior, and (**c**) Erichsen cupping test curves of the as-received and pre-stretched (5%, 10% and 15%) samples.

Figure 4a–c show the room-temperature tensile, strain hardening and Erichsen test curves of the samples (as-received and pre-stretched). The tensile data, including ultimate tensile strength (*UTS*), yield strength (*YS*), uniform elongation (*UE*), total elongation (*TE*), *UTS/YS* ratio, strain hardening exponent (*n*), and Erichsen values are presented in Table 2. The stretched samples exhibited a decreasing trend in tensile properties as compared to the as-received alloy. Among all stretched samples, the 5%-stretched sample showed the lowest YS and UTS (127 MPa, 230 MPa, respectively), but a higher TE of 27%. This enhanced ductility can be attributed to the less homogeneous microstructure received after pre-stretching to 5%. Greater pre-stretching increases the nucleation energy, resulting in an evolution of dynamically recrystallized grains. Accordingly, the increase in YS for 10%- and 15%-stretched samples can be explained based on the transformation from coarser non-recrystallized grains to recrystallized grains as a result of further pre-stretching. Furthermore, the strain hardening exponent (*n*) was evaluated from the uniform plastic deformation area of the true-stress versus true-strain curves (not shown here). It is interesting to notice that the maximum value for strain hardening exponent (0.35) was received for the 5%-stretched sample. For the 10%- and 15%-stretched samples, *n* values (0.23 and 0.21) were lower than as-received and 5%-stretched samples. The higher strain hardening capability of this sample (5%) was additionally confirmed by the UTS/YS ratio, where this ratio was the highest for the 5%-stretched sample as compared to other samples (as-received, 10%- and 15%-stretched), as shown in Table 2. It is already established that the *n* value is the measure for how quickly material gains strength during deformation and is one of the primary factors which control plastic instability during the sheet forming process [33]. Materials with higher *n* values show a greater capacity for being formed (stretched or bent) and also exhibit higher resistance to necking, eventually resulting in enhanced formability. Figure 4b represents the strain–hardening rate curves for the samples (as-received and pre-stretched). It reveals that the rate of strain hardening is higher at the starting stage of deformation and then gradually decreases. The results obtained from Figure 4b are consistent with the *n* values, where, among all the samples, the maximum strain hardening rate was received for 5%-stretched sample. In general, a high strain hardening capability can be reached in Mg alloys by inducing a higher fraction of twins, and this is due to dislocation–twin interactions. In addition, microstructures with less homogeneity exhibit incompatible plasticity, leading to enhanced strain hardening capability [34]. Since the change in the fraction of twins evolved in the samples upon stretching experiments conducted in the present work was less significant as compared to other Mg alloys, the variation in strain hardening behavior recorded for stretched samples (5%-, 10%- and 15%-stretched) cannot be fully figured out based on dislocation–twin interactions. Accordingly, the enhanced strain hardening in the 5%-stretched sample can be related to the less homogeneous microstructure evolved in this alloy as compared to those of 10%- and 15%-stretched samples, as shown by Figure 1 and Figure 2.

Erichsen tests were performed to investigate the impact on stretch formability as a result of the pre-stretching experiment. The Erichsen value can be defined as the depth of the cup in mm required to obtain fracture, which is equal to punch travel distance at the maximum loading force on the load displacement curve during the test, as given by Figure 4c. The average values of 6 mm, 6.9 mm, 4.6 mm and 4 mm were recorded for the as-received, 5%-,10%- and 15%-stretched samples, respectively. It can be noticed that the maximum IE value was received for the sample pre-stretched to 5%, where an improvement of 15% was reached as compared to the as-received sample. As the deformation exceeds beyond 5%, IE values were found to decrease for 10%- and 15%-stretched samples. The significant increase in the formability can be attributed to the less homogeneous microstructure of 5%-stretched sample, which resulted in the higher strain hardening capability observed in this sample as compared to the as-received sample. Generally, the high strain hardening maintains the uniform elongation during the stretching test and reduces the strain localization (related to necking), leading to high stretch formability.

**Figure 5:**
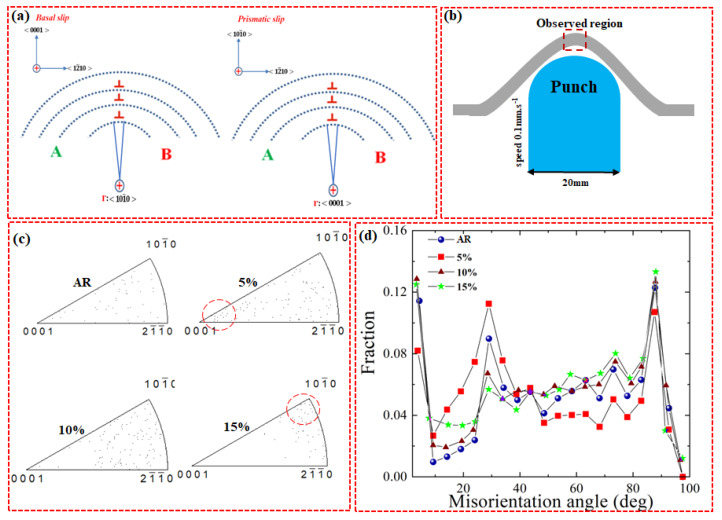
(**a**) Schematic illustration of the relation between in-grain misorientation axis and activated slip systems; (**b**) schematic diagram of the Erichsen test; (**c**) IGMA distribution of the samples (as-received and pre-stretched) after the Erichsen test; (**d**) misorientation profiles of the stretched samples after the Erichsen test.

To determine the slip system, which might be activated during deformation, in-grain misorientation axes (IGMA) analysis was performed on the samples (as-received and pre-stretched) after the Erichsen test. It is already established that IGMA analysis can determine slip-induced lattice rotation and accordingly can identify its rotation axis [35,36]. Generally, the presence of dislocations can bend the crystal which, in turn, can lead to in-grain misorientation (2.5–5°). This bending occurs around an axis (Taylor axis) and rotates the crystallographic planes about that axis (Figure 5a). Hence, the dominant slip system in the deformed sample can be determined by comparing the Taylor axis of slip system to the IGMA. For instance, a misorientation axis close to $\langle 10\bar{1}0\rangle$ can be induced by activation of <a> basal slip while <a> prismatic slip is indicated by a misorientation axis close to 〈0001〉. To measure the IGMA of the samples (as-received and pre-stretched) after the Erichsen test, the top region of the stretch-formed sample was analyzed as schematically shown in Figure 5b. Figure 5c shows the IGMA analysis performed on the samples (as-received and pre-stretched) after the Erichsen test. It can be seen that the IGMA for the 5%-stretched sample is mostly concentrated along 〈0001〉, which is indicative of the activation of prismatic slip. On the other hand, IGMA distributions for the 10%- and 15%-stretched samples are lying along $\langle 10\bar{1}0\rangle$ and $\langle \bar{1}2\bar{1}0\rangle$, which suggest that basal slip is the dominant deformation mechanism in these samples. Accordingly, the high activity of prismatic slip in 5%-stretched sample is believed to be responsible for its high stretch formability. Figure 5d shows the misorientation profiles of the stretched samples after the Erichsen cupping test. It is clearly seen that the 5% sample shows less fraction of misorientation at 86° (tension twins [37]), indicating that the plastic deformation of this sample (5%) during the Erichsen cupping test was more controlled by the non-basal slip systems. In the present work, the high activity of prismatic slip in this alloy (AZ31-0.5Ca alloy) was explained in a previous work and attributed to the formation of (Mg,Al)_2_Ca particles, which increase the stress needed to activate the basal slip, and as a result, decrease the relative CRSS (τ_Prismatic_/τ_Basal_). In the present work, however, all stretched samples have the same composition. Accordingly, the slightly higher activity of the non-basal slip systems in the 5%-stretched sample during and after the Erichsen test is mainly explained based on the texture characteristics evolved in the sample after the stretching experiments.

## 4. Conclusions

In conclusion, the microstructure, texture, and mechanical properties of AZ31-0.5Ca alloy samples stretched to various conditions were evaluated. It was demonstrated that the formability of AZ31–0.5Ca could be further enhanced by pre-stretching, where 5%-stretched sample showed a 15% improvement in formability as compared to the as-received sample. The significant improvement in formability of the 5%-stretched sample can be mainly attributed to the weakening of basal texture and higher strain hardening capability, which, in turn, is related to the formation of more TTs and a less homogeneous microstructure. In addition, the IGMA analysis of samples (as-received and pre-stretched) after the Erichsen test revealed that the grains selected from the 5%-stretched sample are distributed around <0001>, confirming the slightly higher activity of prismatic slip in 5%-stretched samples.

## Figures and Tables

**Table 1:** The composition in wt.% of AZ31-0.5Ca alloy.

Alloy	Al	Zn	Ca	Mn	Si	Fe	Cu	Mg
AZ31-0.5Ca	3.12	0.76	0.5	0.30	0.023	0.0042	0.0012	Balance

**Table 2:** Room-temperature tensile properties and Erichsen values of 5%-, 10%- and 15%-stretched samples. Erichsen tests were carried out at the punch speed of 0.1 mm/s.

Specimens	YS (MPa)	UTS (MPa)	UE (%)	TE (%)	UTS/YS	n	I.E
As received	147	248	31	38	1.68	0.28	6
5%	127	230	20	27	1.81	0.35	6.9
10%	151	228	15	23	1.50	0.23	4.6
15%	152	223	14	22	1.47	0.21	4.0
